# 88116 Effect of conjugated estrogens and bazedoxifene on glucose, energy and lipid metabolism in obese postmenopausal women

**DOI:** 10.1017/cts.2021.498

**Published:** 2021-03-31

**Authors:** Kara Marlatt, Dragana Lovre, Robbie Beyl, Chandra Tate, Evelyn Hayes, Charles Burant, Eric Ravussin, Franck Mauvais-Jarvis

**Affiliations:** 1 Pennington Biomedical Research Center; 2 Tulane University Health Sciences Center; 3 Baton Rouge General Hospital; 4 University of Michigan

## Abstract

ABSTRACT IMPACT: A short treatment of 8 obese postmenopausal women with conjugated estrogens and bazedoxifene does not alter insulin sensitivity or ectopic fat but increases serum markers of hepatic de novo lipogenesis and production of triacylglycerides. OBJECTIVES/GOALS: Combining conjugated estrogens (CE) with the selective estrogen receptor modulator bazedoxifene (BZA) is a novel, orally-administered menopausal therapy. We investigated the effect of CE/BZA on insulin sensitivity, energy metabolism, and serum metabolome in postmenopausal women with obesity. METHODS/STUDY POPULATION: We conducted a randomized, double-blind, crossover pilot trial, testing the effect of CE/BZA on cardiometabolic health in postmenopausal women. Eight postmenopausal women (age 50-60 y, BMI 30-40 kg/m2) were randomization to an 8-week CE/BZA or placebo treatment separated by an 8-week washout period [NCT02274571]. The primary outcome was insulin sensitivity (hyperinsulinemic-euglycemic clamp), while secondary outcomes included body composition (DXA); resting metabolic rate (RMR); substrate oxidation (indirect calorimetry); ectopic lipids (1H-MRS); fat cell size, adipose and skeletal muscle gene expression (biopsies); inflammatory markers; and serum metabolome (LC/MS). RESULTS/ANTICIPATED RESULTS: CE/BZA had no effect on insulin sensitivity, body composition, ectopic fat, or substrate oxidation, but resulted in a non-significant increase in RMR (basal: p=0.06; high-dose clamp: p=0.08) compared to placebo. CE/BZA increased serum high-density lipoprotein cholesterol. CE/BZA also increased serum diacylglycerol (DAG) and triacylglycerol (TAG) species containing long-chain saturated, mono- and polyunsaturated fatty acids (FAs), and decreased long-chain acylcarnitines. These findings possibly reflect increased hepatic de novo FA synthesis and esterification into TAGs, and decreased FA oxidation, respectively (p<0.05). CE/BZA increased serum phosphatidylcholines, phosphatidylethanolamines, ceramides, and sphingomyelins, possibly reflecting the increase in lipoproteins (p<0.05). DISCUSSION/SIGNIFICANCE OF FINDINGS: A short treatment of postmenopausal women with CE/BZA did not alter insulin action or ectopic fat, but increased markers of hepatic de novo lipogenesis and TAG production. Study limitations include a small sample size and short treatment period. A larger, fully powered study is needed to validate the potential metabolic benefit of combining CE with BZA.

